# The Sticking of N_2_ on W(100) Surface: An Improvement in the Description of the Adsorption Dynamics Further Reconciling Theory and Experiment

**DOI:** 10.3390/molecules28227546

**Published:** 2023-11-11

**Authors:** Maria Rutigliano, Fernando Pirani

**Affiliations:** 1 Istituto per la Scienza e Tecnologia dei Plasmi (ISTP), Consiglio Nazionale delle Ricerche (CNR), Via Amendola 122/D, 70126 Bari, Italy; 2 Dipartimento di Chimica, Biologia e Biotecnologie, Università di Perugia, Via Elce di Sotto 8, 06123 Perugia, Italy; pirani.fernando@gmail.com; 3 Dipartimento di Ingegneria Civile ed Ambientale, Università di Perugia, Via G. Duranti 93, 06125 Perugia, Italy

**Keywords:** long-range interactions, molecular dynamics, sticking, tungsten, surface temperature effect

## Abstract

The adsorption of nitrogen molecules on a (100) tungsten surface has been studied using a new potential energy surface in which long-range interactions are suitably characterized and represented by the Improved Lennard–Jones function. The new potential energy surface is used to carry out molecular dynamics simulations by adopting a semiclassical collisional method that explicitly includes the interaction with the surface phonons. The results of the sticking probability, evaluated as a function of the collision energy, are in good agreement with those obtained in the experiments and improve the already good comparison recently obtained with calculations performed using interactions from the Density Functional Theory method and corrected for long-range van der Waals contributions. The dependence of trapping probability on the surface temperature for a well-defined collision energy has also been investigated.

## 1. Introduction

The adsorption of gaseous species on surfaces is of fundamental interest for many processes useful in different applied fields (see, for instance, Refs. [1,2] and the references therein). For this reason, over the years, the complexity of interacting species and the considered substrate has increased.

The reliability of the dynamics and kinetics of the adsorption process, evaluated by computational methods, relies on the accuracy of the used potential energy surface (PES) controlling the evolution of single collision events. In this regard, over the years, with the concomitant advent of powerful supercomputers, it has been possible to determine the PES using increasingly precise and refined methods [3]. The determination of the interaction driving the collisions in heterogeneous gas–surface systems poses different problems, from both the theoretical and computational points of view, primarily for the fact that the involved PES is multidimensional, being dependent on six coordinates. As a consequence, and also due to the large computational time demanded to assure the convergence of calculated energies, complete, fully dimensional electronic structure calculations have been performed for a few elementary systems, notably those involving diatomic molecules on noble and transition metals [4,5]. Among the proposed approximate schemes, the most promising approach is the Density Functional Theory (DFT), which is accurate near the chemisorption potential well, but less accurate for weak long-range interactions. Such weak components are of non-covalent nature and control the gas–surface trapping (physisorption). Therefore, they promote the formation of the precursor (or pre-reactive) state of basic elementary processes, whose role in molecular dynamics is not trivial to identify. In this paper, we have addressed and tried to solve this last point for N_2_ adsorption on a W(100) surface.

In the last decade, several studies have appeared in the literature [6,7,8] with an aim to explain the steep decrease in the sticking probability, the collision energy increasing up to 0.5 eV, as obtained in the experiments made in the late eighties [9,10,11] for nitrogen molecules impinging a W(100) surface. These experiments, in the same collision energy range, also revealed a different reactivity to the case in which nitrogen molecules impact W(110) [12,13]. Understanding and rationalizing the nitrogen adsorption process on tungsten can help to understand and explain the processes that occur on other metal surfaces of interest in nitrogen industrial processes, the ammonia synthesis in primis, which usually involves the interaction of N_2_ and H_2_, mainly with an iron surface [14].

The authors of Ref. [7] succeeded in significantly improving the previous results of simulations reported in Ref. [15] concerning the behavior of the sticking probability as a function of collision energy for N_2_ on W(100), as obtained in experiments [9,10,11]. This improvement was obtained by adopting a PES determined by DFT calculations, including long-range interactions via the vdW-DF2 functional as implemented in the VASP code [16]. The authors of Ref. [7] ascribe the improvement in comparison with the experimental results to the fact that with the introduction of long-range interactions, the barrier in the entrance channel, revealed in the PES of Ref. [15], disappears.

The same authors investigated the energy dissipation during the adsorption dynamics of nitrogen molecules by the Generalized Langevin Oscillator (GLO) for molecule–surface interaction, whilst the Local Density Friction Approximation (LDFA) is implemented to consider the electron–hole (e–h) pair excitations [8]. From this study, it emerged that the interaction with the surface atoms plays a key role in the reaction energetics, while the e–h excitations rather influence the ratio between dissociative and non-dissociative adsorptions [8].

Recently, we proved that the Improved Lennard–Jones (ILJ) potential function [17] can also be useful and accurate for a proper description of the weak long-range non-covalent interactions between gaseous molecules and surfaces arising from the combination of size repulsion with dispersion attraction and controlling the physisorption state formation [18]. The accuracy of this description is derived from the fact that the adopted parameters relate to the intrinsic chemical–physical properties of the reaction partners. Moreover, by adopting the ILJ potential in conjunction with state-to-state molecular dynamics (MD) simulations based on a semiclassical collisional model [19,20], including the interaction with the surface phonons, treated in a quantum way, we were able to explain and support observations made in molecular beams (MB) experiments [21]. In addition, for each simplest-reference interacting pair, the ILJ function provides an asymptotic dispersion attraction associated with a $C_{6}$ coefficient, from which the atom/molecule–surface dispersion coefficient $C_{3}$ can also be evaluated (see next Section 2 and Section 3).

Therefore, this work aims to apply our computational setup to the N_2_-tungsten system to improve further the comparison between experiment and theory for the sticking probability and, at the same time, to provide an additional contribution to understanding reaction dynamics and the underlying energetics. To do this, we mainly focused on the evaluation of the long-range interaction strength, while also determining the $C_{6}$ and $C_{3}$ coefficients.

We built a new PES by grafting the short-range interaction data, taken from the literature, onto the long-range interaction modeled according to the ILJ potential. Then, we used the obtained PES, given in the proper analytical form, for MD simulations of N_2_ molecules impinging the W(100) surface in the low-collision-energy range. The surface temperature (T_S_) effect on the sticking probability, due to being related closely to the energy exchange with the phonons, explicitly considered in the adopted method, has also been investigated. The proper inclusion of surface phonons produces an agreement improved with the experimental results, whilst the correct treatment of the long-range interaction determining the precursor state suggests a new picture for the reaction dynamics.

Thus, the paper is organized in the following way: Section 2 focuses on the results obtained for the long-range interaction potential for the three active sites on the W(100) surface and provides the dispersion coefficients, which are also compared with the results available in the literature; in the same section, the results obtained for the sticking probability as a function of collision energy and surface temperature will be presented and discussed. Section 3 provides all basic details of the methods employed to carry out MD simulations and to formulate the PES. Some conclusions are drawn in Section 4.

## 2. Results and Discussion

### 2.1. The ILJ Long-Range Potential of N and N_2_ Interacting with W(100)

**Figure 1:**
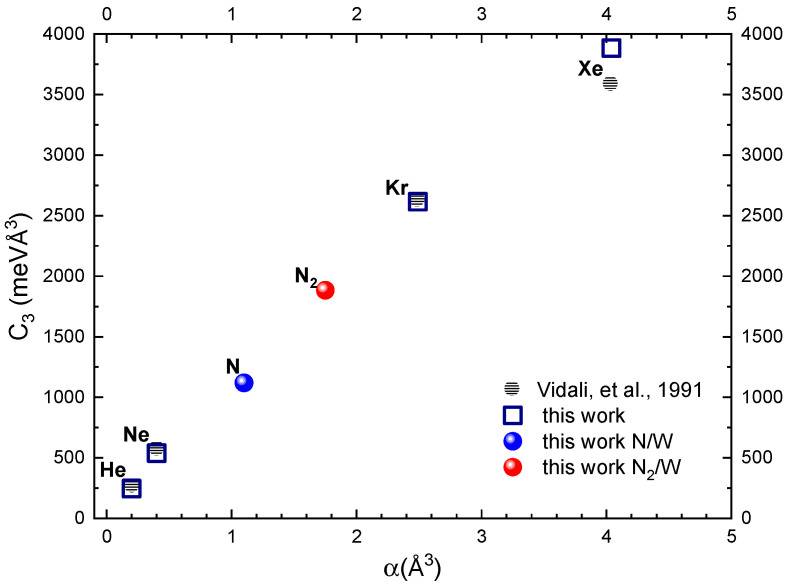
$C_{3}$ coefficients calculated for rare gases and nitrogen (atomic and molecular) as a function of polarizability (α) of the gaseous species interacting with W(100) in comparison with the data of Ref. [23]. The polarizability values of rare gas atoms and N_2_ are taken from Ref. [24] and that of N from Ref. [25]. Note that while the relative $C_{3}$ values vary almost linearly with the rare gas atoms’ polarizability, their absolute value also depends on the effective polarizability of W atoms bounded in the surface.

Details on the ILJ formulation, representing the weak long-range non-covalent part of each interaction potential, are summarized in the Section 3. Table 1 provides values of the basic ILJ potential parameters and of related$\;C_{3}$ and $C_{6}$ dispersion coefficients. By exploiting high-resolution gas-phase MB experiments, it has been demonstrated [17] that the $C_{6}$ coefficients, representative of the asymptotic behavior of the ILJ potential, are in good agreement with the results obtained from other experimental sources, semiempirical predictions and/or ab initio calculations. In this section, the focus is on the asymptotic part of the PES affecting the gas–surface trapping and on the comparison between the C_3_ coefficients, obtained in an internally consistent way from the ILJ $C_{6}$ coefficients, with the results available in the literature. In particular, the parameters reported in Table 1 have been evaluated, according to Refs. [17,22], by exploiting the polarizability of the single, isolated N atom (1.1 Å^3^) and the effective polarizability value of N in N_2_ (0.88 Å^3^). We also estimated the value of the effective polarizability of each W atom bound in the surface equal to 2.9 Å^3^ and considered an atomic surface density (ρ) equal to 0.0632 atom/Å^3^. In Figure 1, the $C_{3}$ coefficient for the nitrogen atom and molecule is reported as a function of their polarizability (α) in comparison with the values, determined with the same method, for rare gases interacting with W(100). In the same figure, the $C_{3}$ coefficients for the interaction of rare gases with W(100), obtained with different methods [23], are reported for a useful comparison. Looking at the plot, we can infer both the linear correlation between the $C_{3}$ coefficient and α, and a good comparison between the data reported in the literature and those calculated in this work for rare gases. Moreover, the right placement of points for N and N_2_ in the plot makes us confident about the parameters used to obtain the long-range potential of interest for the present investigation.

**Figure 2:**
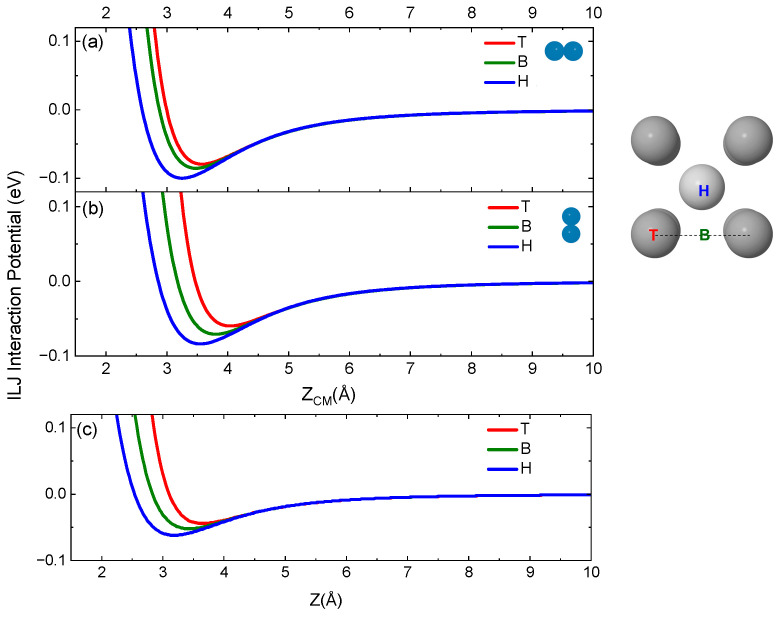
ILJ potential for N_2_ impinging with its CM on T, B and H sites and with molecular axis being (**a**) parallel and (**b**) perpendicular to the surface plane. (**c**) ILJ potential for nitrogen atom impinging on the three sites. On the right side of the figure is the top view of the unit cell of W(100) on which the three sites on the surface are located. The color of the curves is associated with that of the site label. In the plot, Z defines the component of R along the normal direction and Z = 0 corresponds to the surface first layer.

In Figure 2, the ILJ potential for the three active sites on the surface, top (T) on a W atom, bridge (B) between two adjacent W atoms and hollow (H) in the center of the bcc unit cell, are reported for N_2_ impinging with the center of mass (CM) on the given site for two different orientations of the molecular axis with respect to the surface plane, parallel and perpendicular. In the same Figure, the ILJ potential for the N atom is also provided for the three sites.

From Figure 2, it emerges that the H site is the most attractive and that the minimum is placed at shorter distances from the surface compared with the other sites. Furthermore, for both atom and molecule, the physisorption well depth decreases with the sites according to the H > B > T order. Moreover, for the same site, the molecule in parallel configuration undergoes greater attraction, with the well depth being close to 0.1 eV for the H site.

### 2.2. Potential Energy Surface Determination

The complete reactive PES has been obtained as a sum of pairwise interactions, according to the consolidated functional form already used for the study of several molecule–surface systems [21] and given in Equation (1).

(1) $${V(r,R)=V}_{N2}(R)\times fsw(r)+V_{N}(R)\times (1-fsw(r))$$

V_N2_(R) and V_N_(R) represent (nitrogen molecule)– and (nitrogen atom)–surface interaction potentials, respectively. R is the distance of impinging species from the surface. The weight function fsw(r) switches the interaction potential between V_N2_ and V_N_ as r, the interatomic distance between the atoms in the molecule, increases and is given by

(2) $$fsw\left(r\right)=-0.5\times \left(\left(\tanh\left(1.65\times r-3.7\right)\right)-1.0\right)$$

This function has been chosen to provide quick switching between 1 and 0, preserving the values of V_N2_ and V_N_ for r lower and higher, respectively, than the intramolecular distance considered critical for N_2_ molecule dissociation (3 ÷ 3.5 Å).

V_N2_(R) has been obtained by combining the interaction potential on the three different sites (see Figure 2) on the surface through a switch function fswhbt, given in Equation (4):

(3) $$V_{N2}\left(R\right)=\sum_{j=1}^{2}\sum_{i=1}^{N_{at}}\left[V_{H}\times fswhbt+(1-fswhbt)\times (V_{T}\times (1-fswbt)+V_{B}\times fswbt)\right]$$

the sum on j runs on the two atoms in the molecule while that on i runs on the atoms N_at_ in the assumed surface model lattice.

(4) $$fswhbt={\left(FA\right)}^{2}\times FB$$

with

$$FA=\cos\left(1.5\times \pi +\pi \times \frac{X_{g}}{a}\right)\;and\;FB=|\sin\left(\pi \times \frac{Y_{g}}{a}\right)|$$

X_g_ and Y_g_ are the molecule CM coordinates on the X–Y plane of the assumed reference frame; a is the lattice constant of W(100), equal to 3.165 Å.

fswbt is another switching function to discriminate between B and T sites and has the following functional form:

(5) $$fswbt=\left|F1+F2\right|\;with\;F1=\sin\left(\pi \times \frac{X_{g}}{a}\right)\;and\;F2=\sin\left(\pi \times \frac{Y_{g}}{a}\right)$$

The interaction potential for the H site is determined as a pure ILJ potential according to the data available in the literature [6,26] for both orientations of N_2_ molecule impinging to the surface. Instead, for sites T and B, the ILJ potential was added to the fitting value of the potential determined in Refs. [6,26] for the incidence of N_2_ in a perpendicular and parallel configuration, respectively. In fact, the authors of both papers determine the interaction potentials for distances up to 4.0 ÷ 5.0 Å, too short to control the asymptotic behavior at longer distances, responsible for the formation of the precursor state.

For V_T_ and V_B_, the expression, as a function of the CM distance along the normal to the surface of the incident molecule, is

(6) $$V_{S}=\left\{\begin{array}{ll} D_{S}\times e^{-b_{S}\times \left(R-R_{S}\right)}\times \left(e^{-b_{S}\times \left(R-R_{S}\right)}-2\right)+x_{S} & \;\;\;Z_{g}<Z_{S}\\ V_{ILJ} & \;\;\;Z_{g}\geq Z_{S} \end{array}\right.$$

where $Z_{g}$ is the Z coordinate of molecule CM in the assumed reference frame.

The subscript S from here can be T or B. Note that the switch between the two functional expressions of the potential occurs as a function of Z, the component along the normal direction to the X–Y plane of R.

The general expression for the parameter P (P generally indicating the$\;D_{S}$, $b_{S}$ and $R_{S}$ Morse function parameters; $x_{S}$ being the additional long-range correction factor; and $Z_{S}$ denoting the switch value for Z) depends on the site and orientation of the molecular axis (θ) relative to the surface plane. Then, its analytic form is P = A + B (1 − cos(θ)) and the values of the constants for each site are given in Table 2.

**Figure 3:**
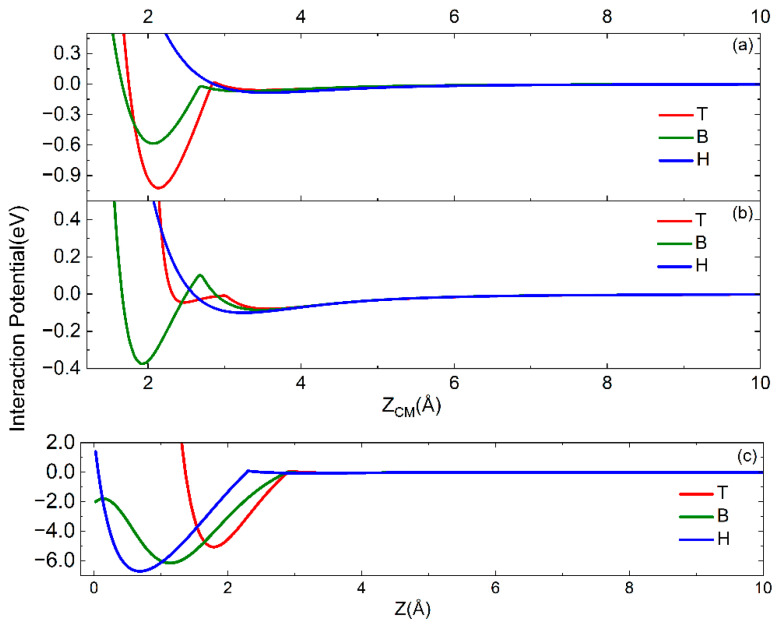
Interaction potentials for N_2_ impinging with CM on T, B and H sites and with molecular axis (**a**) parallel and (**b**) perpendicular to the surface plane. (**c**) Interaction potential for nitrogen atom impinging on the three sites. The correspondence between the colors of the curves and the sites is the same as in Figure 2.

The complete interaction potential for the N_2_ molecule impinging with perpendicular and parallel orientation of its molecular axis and with CM on the three active sites on the W(100) surface is reported in Figure 3a,b.

The interaction potential for atomic nitrogen for short distances has been obtained by fitting the results of Refs. [6,27]. The analytical form for V_N_ is the same as Equation (3). For atomic nitrogen also, the interaction potential for the H site is obtained as the sum of short- and long-range contributions. The terms corresponding to the different sites are given similarly by the expression of Equation (6), while the corresponding parameters are reported in Table 3. The behavior of the obtained potential is reported in Figure 3c.

Looking at Figure 3, it is clear that the PES for the interaction of N and N_2_ on W(100) results strongly corrugated. It is interesting to note that the molecular approach towards well-defined surface sites can introduce a small barrier for the transition from the physisorption well to the chemisorption well.

### 2.3. The Sticking Probability for N_2_ Interacting on W(100)

**Figure 4:**
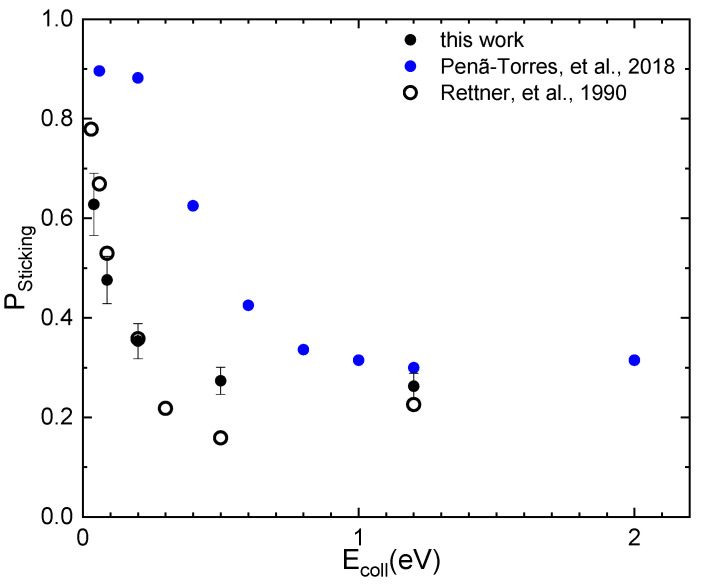
Sticking probability (P_Sticking_) for N_2_ interacting in normal direction on W(100) as a function of collision energy in comparison with results of latest calculations [7] and of experiments [10]. T_S_ = 300 K.

The values of sticking probability as a function of N_2_ collision energy (E_coll_) obtained by MD simulations are reported in Figure 4 for T_S_ = 300 K in comparison with experimental data [10] and the results of previous simulations [7]. The values reported in the plot are obtained by including both dissociative and molecular adsorption events. Looking at the plot, it appears that the comparison with experimental results is improved significantly with respect to the comparison with the results of Ref. [7], mainly for the lower collision energies (E_coll_ < 0.2 eV). In particular, in this energy range, the slope with which the sticking probability (P_Sticking_) decreases is almost the same as that of the experimental data. These findings, in comparison with those of Ref. [7], could indicate a different mechanism in the interaction dynamics due to the adoption of a dissimilar long-range interaction potential and/or that the treatment of the interaction with the phonons of the surface adopted here is able to describe better the energy exchanges between the incident species and the surface.

**Figure 5:**
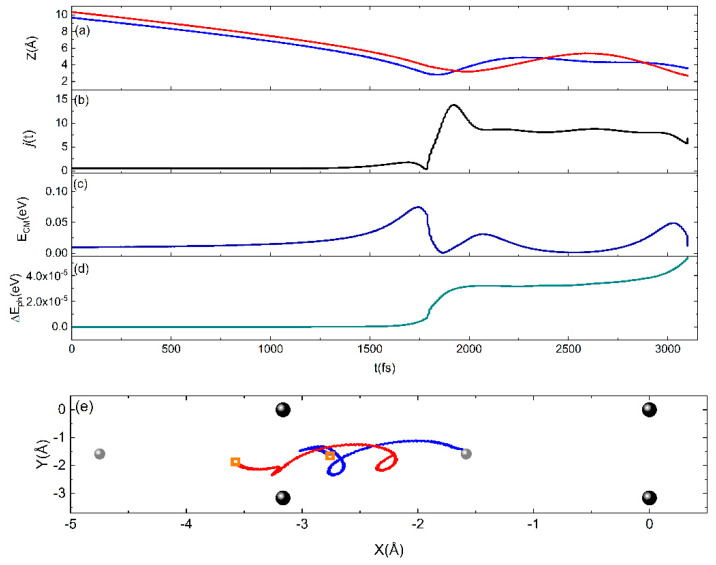
A typical sticking trajectory for E_coll_ = 0.04 eV. Time evolution of (**a**) the coordinate of the two atoms (lines blue and red) in the N_2_ molecule, along the Z normal direction, which ends with the adsorption; (**b**) the rotational state j; (**c**) the center of mass translational energy (E_CM_); (**d**) the energy exchanged with the surface phonons; and (**e**) the diffusion motion in the X–Y plane, of assumed reference frame, of the trajectory of two atoms (blue and red lines) in the N_2_ molecule (orange squares indicate the starting point of each trajectory). The W atoms on the first (big black spheres) and second (small grey spheres) layers are also reported.

To settle the matter, an accurate analysis of the trajectories has been carried out to highlight the energy exchanges occurring between the internal degrees of the molecule and the surface. The analysis revealed that, for low collision energies and with the molecule in the lowest internal state, the interaction is strong mainly when the molecules approach the surface by moving with a cartwheel-type internal motion. In this case, the rotational excitation mechanism, being the most efficient, favors the trapping that, in turn, can promote bounces on the surface, also contributing to the desynchronization of the motion of the two N atoms in the molecule. This behavior has already been observed in the interaction of nitrogen molecules with a graphite surface [21] and related to the surface phonons. In addition, as observed in previous studies for the interaction of molecules with graphite and silica surfaces [20,21,28], the molecule approaching the surface is first slightly accelerated and then undergoes a strong deceleration while transferring energy to the rotational motion. Consequently, a strong increment of the rotational number occurs to which the energy coming from the phonons of the surface also contributes, albeit with a smaller amount. The occurrence of such a trapping mechanism has already been advanced in Ref. [29] to explain the adsorption of hydrogen molecules on a cold Cu surface. Therefore, in light of the above, the obtained results for P_Sticking_ can be explained in terms of a dynamic steering mechanism. In our picture of interaction dynamics, the rotational excitation counteracts the steering that favors the molecule path towards a direct dissociation and molecules remain trapped on the surface. In the past, the role of rotational effect in the adsorption of molecules on metals has been observed in the MB experiments [30,31] and studied in Refs. [32,33]. In addition to the dynamic steering, the results for P_Sticking_, for low collision energies, can also be ascribed to either the physisorption well appearing in the assumed PES and having a depth greater than or, at most, of the same order (see Section 2.2) as E_coll_, which prevents the molecule from being immediately scattered in gas-phase, and/or to the existence of a barrier that can be higher than the collisional energy. The dynamic mechanism just described can be observed in Figure 5, which shows one of the trajectories ending with molecular adsorption for E_coll_ = 0.04 eV.

Thus, the ILJ potential used to treat the long-range interactions exploited by a state-to-state model, including the coupling with the surface phonon motion, is able to highlight molecular excitation governing the process dynamics and, with respect to previous calculations, provides results much closer to those resulting from experimental measurements.

**Figure 6:**
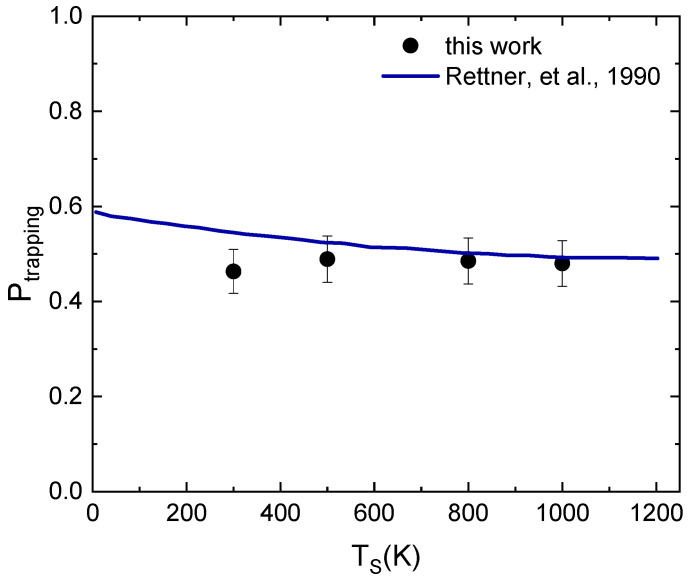
Trapping probability (P_trapping_) for N_2_ interacting on W(100) as a function of surface temperature (T_S_) at E_coll_ = 0.088 eV in comparison with results obtained in Ref. [10].

The role of coupling of the N_2_ molecule with phonons is further extolled by considering, at a selected collision energy, the dependence of the trapping probability (P_trapping_) on T_S_. P_trapping_ represents the probability for a molecule to become trapped on the surface or eventually, after a while spent bouncing on the latter, to be scattered in the gas-phase. We did this, and in Figure 6, we report the results of our study in comparison with those reported in Ref. [10], obtained by adopting a hard cube model with parameters chosen to provide results consistent with experimental measurements. Looking at Figure 6, we can conclude that, even in this case, there is a very good agreement, considering the error of our results and the approximations and parametrization of Ref. [10] to obtain the line in the plot based on experimental measurements.

The plot in Figure 6 also suggests a weak dependence on the surface temperature of P_trapping_, which appears to be more strongly correlated to the collision energy of the molecule and the energy exchanges occurring during the interaction. Such almost non-dependence on the surface temperature of P_trapping_ can perhaps be attributed to the mass mismatch between the incident species and the substrate.

## 3. Methods

The computational setup, used to describe the collisions of atomic and molecular nitrogen with W(100) surface, is based on a state-to-state semiclassical collision method, derived and completely exposed in Ref. [19], which has been used in the past for the description of surface processes; therefore, it has already been extensively described elsewhere [34] and more recently in Ref. [20]. For this reason, in the following, only a brief summary of the most important and specific features will be provided.

The method consists of three different operational steps: (1) determination of 3D surface model structure and corresponding surface phonon dynamics; (2) building up of PES for the reaction under study; and (3) propagation of a sufficiently large number of classical trajectories in the adopted framework [19,20].

The 3D surface model, consisting of 255 atoms disposed on six layers, is the one defined in Ref. [34] for which we determined the phonon dynamics by solving the time-dependent Schrödinger equations of motion under the harmonic oscillator approximation—that is, assuming that a set of M = 3*N*_at_ − 6 independent harmonic oscillators are perturbed by a linear force exerted between the species approaching the surface from the gas-phase and the solid substrate [19]. Details on the bulk potential and the density of phonon states can be found in Ref. [34].

The PES has been built by adding to the short-range potential (described in Section 2.2) the long-range interactions according to the ILJ potential [17] given by

(7) $$V_{ILJ}\left(R\right)=\varepsilon \left[\frac{m}{n\left(R\right)-m}{\left(\frac{R_{m}}{R}\right)}^{n(R)}-\frac{n\left(R\right)}{n\left(R\right)-m}{\left(\frac{R_{m}}{R}\right)}^{m}\right]$$

with

(8) $$n\left(R\right)=\beta +4{\left(\frac{R}{R_{m}}\right)}^{2}$$

The first term of Equation (7) describes the separation distance dependence of the size repulsion, while the second one is that of the dispersion attraction. The parameters ε and $R_{m}$, which represent the potential well depth and its location, respectively, for each considered pair, define at each R the strength of both terms in Equation (7). β is an additional parameter depending on the “hardness” of the two partners. For the neutral–neutral interactions, as in the present ones, m = 6 must be used: in these cases, the ILJ function provides for each interacting pair an asymptotic dispersion attraction contribution associated with a partial $C_{6}$ coefficient, defined as$\;C_{6}=\varepsilon \times R_{m}^{6}$. The combination of all partial $C_{6}$ coefficients determines the value of the global atom/molecule–surface attraction coefficient $C_{3}$, which controls the capture efficiency and the formation of the precursor state of many gas–surface elementary processes. The C_3_ coefficient is obtained through the well-known relationship (see Ref. [21] and references therein) that binds it to the $C_{6}$ coefficient:

(9) $$C_{3}=\frac{\pi \times C_{6}\times \rho }{6}$$

The dynamics of the N_2_ molecule interacting with W(100) surface is followed by solving self-consistently the relevant 3D Hamilton’s equations of motion with those of the lattice phonons, under given initial conditions:

(10) $$\dot{R_{i}}=\frac{\partial H}{\partial P_{i}};\;\dot{P_{i}}=-\frac{\partial H}{\partial R_{i}}$$

$P_{i}$ is the momentum of atom i having mass m_i_ and H is the Hamiltonian for a diatomic molecule impinging on a surface, given by

(11) $$H=\frac{1}{2}\sum_{i}\frac{P_{i}^{2}}{m_{i}}+V_{N_{2}}\left(r\right)+\Delta E_{ph}+V_{eff}(t,T_{S})$$

with$\;V_{N_{2}}(r)$ being the N_2_ intramolecular interaction potential and $V_{eff}(t,T_{S})$ the effective potential of mean field type, depending on time and surface temperature, formulated as

(12) $$V_{eff}\left(t,T_{S}\right)=V_{0}+\sum_{k}V_{K}^{\left(1\right)}\eta_{k}(t)$$

where $V_{0}$ is the “static” interaction potential, given by Equation (1), between the atoms in the gas-phase and the lattice atoms in their equilibrium positions. $V_{k}^{\left(1\right)}=\partial V(r,R)/\partial Q_{k}{|}_{eq}$ is the linear driving force exerted on each k-th phonon mode Q_k_.$\;\eta_{k}$ are the “phonon excitation strengths” given in terms of the Fourier components $I_{i,k}$ of the external force:

(13) $$\eta_{k}\left(t\right)=-\int dt^{\prime }{\left(\hbar \omega_{k}\right)}^{-1}\frac{d}{d\rho_{k}}\left(\Delta E_{k}^{+}+\Delta E_{k}^{-}\right)\left[I_{c,k}\left(t^{\prime }\right)\cos\left(\Theta_{k}\left(t^{\prime },\omega_{k}\right)\right)+I_{s,k}\left(t^{\prime }\right)\sin\left(\Theta_{k}\left(t^{\prime },\omega_{k}\right)\right)\right]$$

(14) $$I_{c,k}=\int_{-\infty }^{+\infty }dtV_{k}^{\left(1\right)}\left(R\left(t\right)\right)\cos\left(\omega_{k}t\right)$$

with $\Theta_{k}\left(t,\omega_{k}\right)\approx \omega_{k}t$, $\omega_{k}$ being the frequency of the k-th phonon mode.$\;\Delta E_{k}^{\pm }\;$is the energy exchanged between the impinging molecule and the solid substrate due to the phonon creation and phonon annihilation processes. The energy exchanged with the phonons can be obtained directly from the transition probabilities for the excitation/deexcitation phonon processes [19].

The rotational and vibrational states of N_2_ molecules, assumed as a Morse oscillator [35], were analyzed in terms of the action-angle variables using the semiclassical quantization rules [36]. Therefore, the roto-vibrational states were determined as continuous variables, as we are unable to predict some features caused by quantum effects and selection rules.

In this computational framework, we took the nitrogen molecule impinging along the normal to the surface planes starting from a distance of 10 Å and with collision energy in the range [0.04–1.2] eV. The starting distance was chosen as a compromise between reasonable calculation times for trajectory propagation and the need to consider the effects of long-range interactions. For each E_coll_ value, we propagated 30,000 trajectories, while T_S_ was fixed to 300 K. The initial coordinates of impinging species were randomly generated at the beginning of each trajectory, in an aiming area coinciding with the unit cell. The molecule CM impinges with polar angle θ = 0°, defining the selected normal approach, and azimuthal angle (ϕ) of the molecular axis was randomly chosen at the beginning of each trajectory. We can describe and follow the different elementary surface processes occurring when the N_2_ molecule impinges on the surface, as the assumed PES is reactive. The impact of a molecule on a surface can give rise to scattering (elastic or inelastic), adsorption of both atoms, desorption of just one atom with the other adsorbed on the surface or desorption of both atoms as a molecule or separated.

The criteria adopted in the analysis of trajectories, leading to the assignment of a given trajectory to one of the listed reaction channels, are similar to those used in Ref. [28]. So, molecular scattering occurs if, after the interaction with the W(100) surface, the intramolecular distance (r) is lower than the distance of dissociation for the N_2_ molecule and, at the same time, the distance between molecule CM and the surface is larger than 8.0 Å. On the contrary, if after the interaction with the tungsten surface the distance between N_2_ CM and the surface is comparable to or smaller than the distance (≈5.0 Å) at which the potential approaches its asymptotic value, and r is lower than the dissociation distance, the molecule is considered trapped in the physisorption well. Further, a second “energy” criterion can be followed according to which the molecule is adsorbed when the energy available to escape from the potential well is less than the effective potential, accounting for the interaction with the surface phonons.

The trapped molecule can be subject to a steering process that can produce one of the processes listed above.

## 4. Conclusions

This study presents a new PES controlling the collision dynamics of N_2_ molecules impinging on the W(100) surface. The long-range interaction components, defining the asymptotic behavior of the PES, have been properly characterized and represented by an ILJ function. MD simulations, performed with a semiclassical collisional model, have been exploited to characterize basic details of the collision dynamics, including its dependence on collision energy, ranging from sub-thermal up to hyper-thermal conditions. In particular, we focused on the sticking probability, with its dependence on the collision energy; on the trapping probability, with its dependence on the surface temperature for a selected E_coll_; and on their comparison with the corresponding experimental determinations. This study proves that the asymptotic part of the interaction plays a crucial role in the molecular interaction dynamics since defining all relevant features of the precursor state controls the dynamics of basic phenomena occurring at the gas–surface interphase. Furthermore, the description adopted here for long-range forces is found to be more suitable than that obtained by using DFT methods with appropriate corrections.

## Figures and Tables

**Table 1:** ILJ adopted parameters and related dispersion coefficients. Note that ε, R_m_ and $C_{6}$ represent, respectively, the potential well, the minimum location and the dispersion attraction coefficient of each weak interacting effective atom-effective atom pair. β, related to the hardness of the partners, is a parameter defining the shape of the potential well. The $C_{3}$ coefficient, defining the long-range atom/molecule–surface dispersion attraction, is proportional to $C_{6}$ multiplied by the atomic surface density (for further details, see the Section 3).

Interaction	ε(meV)	$R_{m}$(Å)	β	$C_{6}$(meV Å^6^)	$C_{3}$(meV Å^3^)
N(N_2_)–W	6.23	4.07	6.3	28,452	941.5
N–W	7.40	4.07	7.0	33,635	1119

**Table 2:** Constant for the potential parameters for T and B sites for N_2_ impacting on W(100).

Site	$D_{S}$(eV)		$b_{S}$(Å^−1^)		$R_{S}$ (Å)		$x_{S}$ (eV)		$Z_{S}$ (Å)	
	A	B	A	B	A	B	A	B	A	B
T	0.693	−0.675	1.16	3.59	2.54	−0.01	0.0079	−0.0079	2.95	0.07
B	0.390	−0.155	1.25	1.1	2.89	−0.63	0.0065	−0.0057	2.72	−0.03

**Table 3:** Constants for the potential parameters of T, B and H sites for N impacting on W(100).

Site	$D_{S}$ (eV)	$b_{S}$ (Å^−1^)	$R_{S}$ (Å)	$x_{S}$ (eV)	$Z_{S}$ (Å)
T	3.845	1.45	1.97	0.014	2.88
B	2.35	1.75	2.08	0.0051	2.95
H	1.69	1.50	2.42	0.018	2.30

## Data Availability

The data presented in this study are available on request from the corresponding author.
